# Application of Paramagnetic NMR-Validated Molecular Dynamics Simulation to the Analysis of a Conformational Ensemble of a Branched Oligosaccharide

**DOI:** 10.3390/molecules17066658

**Published:** 2012-05-31

**Authors:** Ying Zhang, Sayoko Yamamoto, Takumi Yamaguchi, Koichi Kato

**Affiliations:** 1 Institute for Molecular Science and Okazaki Institute for Integrative Bioscience, 5-1 Higashiyama, Myodaiji, Okazaki 444-8787, Japan; Email: yzhang@ims.ac.jp (Y.Z.); sayokoy@ims.ac.jp (S.Y.); takumi@ims.ac.jp (T.Y.); 2 Department of Functional Molecular Science, the Graduate University for Advanced Studies, 5-1 Higashiyama, Myodaiji, Okazaki 444-8787, Japan; 3 Graduate School of Pharmaceutical Sciences, Nagoya City University, 3-1 Tanabe-dori, Mizuho-ku, Nagoya 467-8603, Japan; 4 The Glycoscience Institute, Ochanomizu University, 2-1-1 Ohtsuka, Bunkyo-ku, Tokyo 112-8610, Japan; 5 GLYENCE Co., Ltd., 2-22-8 Chikusa, Chikusa-ku, Nagoya 464-0858, Japan

**Keywords:** oligosaccharide, molecular dynamics, NMR spectroscopy, lanthanide, pseudocontact shift, ganglioside

## Abstract

Oligosaccharides of biological importance often exhibit branched covalent structures and dynamic conformational multiplicities. Here we report the application of a method that we developed, which combined molecular dynamics (MD) simulations and lanthanide-assisted paramagnetic NMR spectroscopy, to evaluate the dynamic conformational ensemble of a branched oligosaccharide. A lanthanide-chelating tag was attached to the reducing end of the branched tetrasaccharide of GM2 ganglioside to observe pseudocontact shifts as the source of long distance information for validating the conformational ensemble derived from MD simulations. By inspecting the results, the conformational space of the GM2 tetrasaccharide was compared with that of its nonbranched derivative, the GM3 trisaccharide.

## 1. Introduction

Oligosaccharides that modify proteins and lipids play a crucial role in cell-cell communications, viral infections, and the fate determination of proteins, serving as mediators in various molecular recognition events on cell surfaces and in intracellular environments [1,2,3]. To interpret biological messages carried by oligosaccharides, it is essential to determine their three-dimensional (3D) structures at the atomic resolution. Oligosaccharides possess significant degrees of freedom in internal motion, which endow them with conformational adaptability upon interacting with various protein molecules as binding targets [4,5]. Another unique structural feature of oligosaccharides is their branching with multiple modes of linkages in contrast to DNA, RNA, and protein molecules.

NMR spectroscopy is a useful tool for the conformational analysis of oligosaccharides in solution [6,7,8,9]. However, it is not feasible to determine the dynamic conformation of oligosaccharides solely on the basis of local conformational restraints derived from nuclear Overhauser effects (NOEs) and scalar couplings. Recently, paramagnetic NMR techniques have been developed to provide long distance information for the characterization of oligosaccharide conformations at the atomic level [10,11,12]. These techniques have been successfully applied to the conformational analysis of the disaccharides *N*, *N′*-diacetylchitobiose [10] and lactose [11], which correspond to the rigid core of *N*-linked oligosaccharides and the flexible innermost junction of ganglioside sugar moieties, respectively. In particular, we have demonstrated that large-scale molecular dynamics (MD) simulations in conjunction with lanthanide-assisted NMR spectroscopy enabled the atomic description of a dynamic ensemble of oligosaccharide conformations in solution using the trisaccharide of ganglioside GM3 αNeu5Ac-(2-3)-βGal-(1-4)-βGlc as the model molecule [13]. In this approach, a metal-chelating tag is covalently attached to the reducing end of the trisaccharide for observing pseudocontact shifts (PCSs), which depend on the relative positions of the individual atoms with respect to the lanthanide ion coordinated at the tag. The observed PCS values are compared with those back-calculated from the MD-derived conformational ensemble of the trisaccharide to validate the simulation. This method is useful in evaluating the dynamic conformational ensembles of oligosaccharides, considering minor conformers that are barely detected by other experimental techniques.

We herein attempted to apply this approach to the conformational characterization of branched oligosaccharides. We used the GM2 tetrasaccharide βGalNAc-(1-4)-[αNeu5Ac-(2-3)]-βGal-(1-4)-βGlc as a test molecule, which possesses an additional GalNAc branch in comparison with the GM3 trisaccharide. By comparing the structural data between the GM2 and GM3 sugar chains, we will discuss how the branch affects the conformation of the rest of the molecule.

## 2. Results and Discussion

### 2.1. MD Simulations and PCS Analyses of the GM2 Tetrasaccharide

**Figure 1:**
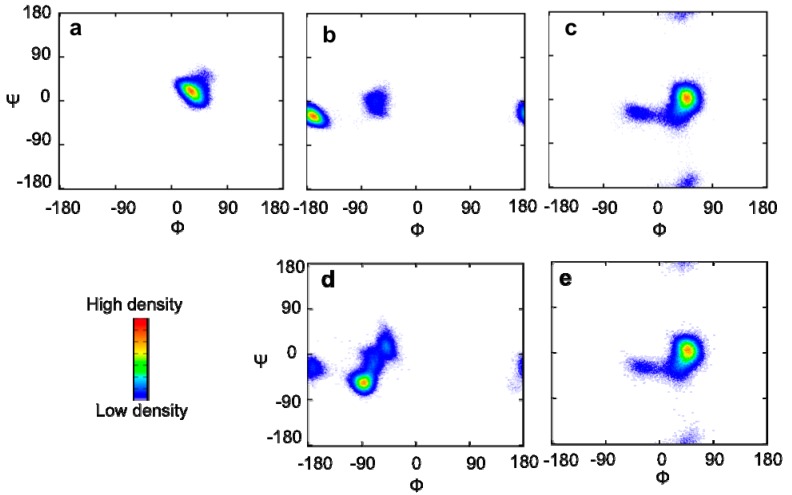
Torsion angle density maps of the combined MD trajectory of (**a**, **b**, and **c**) GM2 tetrasaccahride and (**d** and **e**) GM3 trisaccahride [13]. (**a**) The GalNAc-Gal, (**b** and **d**) the Neu5Ac-Gal, and (**c** and **e**) the Gal–Glc linkages. The d and e parts of this figure were reproduced by permission of The Royal Society of Chemistry. The definitions of Φ and ψ were used for the GalNAc–Gal and the Gal–Glc linkages, Φ = H1–C1–O'4–C'4 and ψ = C1–O'4–C'4–H'4, and the Neu5Ac–Gal linkage, Φ = C1–C2–O'3–C'3 and ψ = C2–O'3–C'3–H'3.

All-atom MD simulations with the GLYCAM_06 force field [14] were employed to capture the conformational dynamics of the sugar moiety of GM2. Ten MD simulations were performed in explicit water for 12 ns at 300 K to generate the atomic coordinates of the tetrasaccharide. After excluding the first 2 ns of the trajectories, all MD runs were combined, and the glycosidic torsion angles were monitored (Figure 1). The torsion angles of the GalNAc-Gal glycosidic linkage of this tetrasaccharide populated one cluster with averaged angles (Φ, ψ) = (30° ± 12°, 17° ± 13°). In contrast, two clusters of the torsion angles of the Neu5Ac-Gal linkage, (Φ, ψ) = (−174° ± 11°, −32° ± 11°) and (−69° ± 10°, −6° ± 14°), and three clusters of the Gal-Glc linkage, (Φ, ψ) = (−34° ± 15°, −32° ± 17°), (40° ± 11°, −4° ± 20°) and (37° ± 21°, −167° ± 19°), were observed. 

**Scheme 1:**
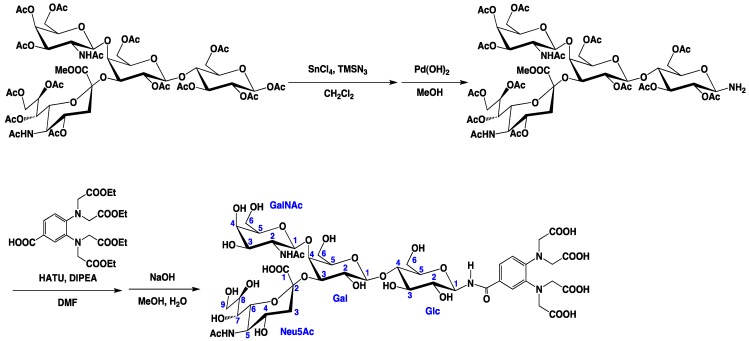
Paramagnetic tagging of the GM2 tetrasaccharide.

To evaluate the conformation dynamics of the GM2 tetrasaccharide, PCS analyses of the oligosaccharide were performed. A paramagnetic lanthanide tag was introduced to the GM2 tetrasaccharide, as shown in Scheme 1. The reducing terminus of the tetrasaccharide was aminated in good yield by selective azidation and subsequent reduction reactions, and then covalently attached to a phenylenediamine derivative. 

**Figure 2:**
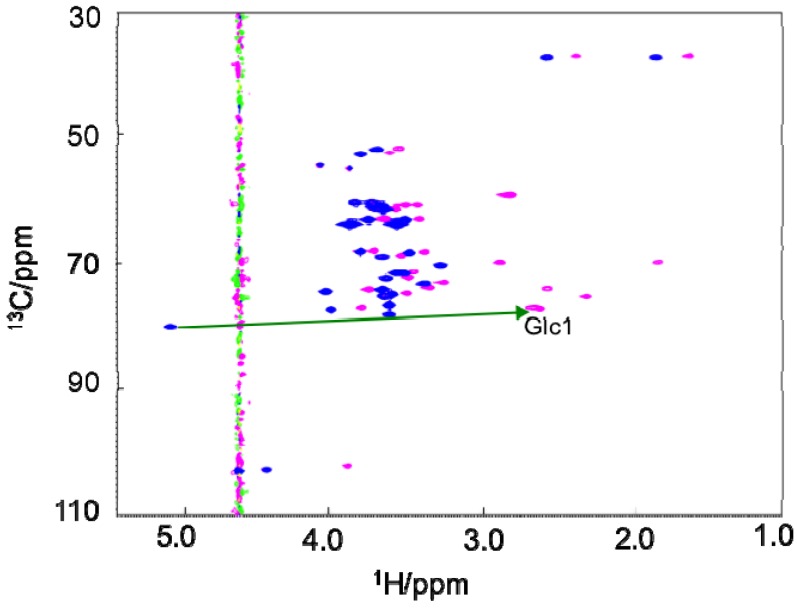
^1^H-^13^C HSQC spectra of the GM2 tetrasaccharide tagged with Tm^3+^ (magenta) and La^3+^ (blue).

**Figure 3:**
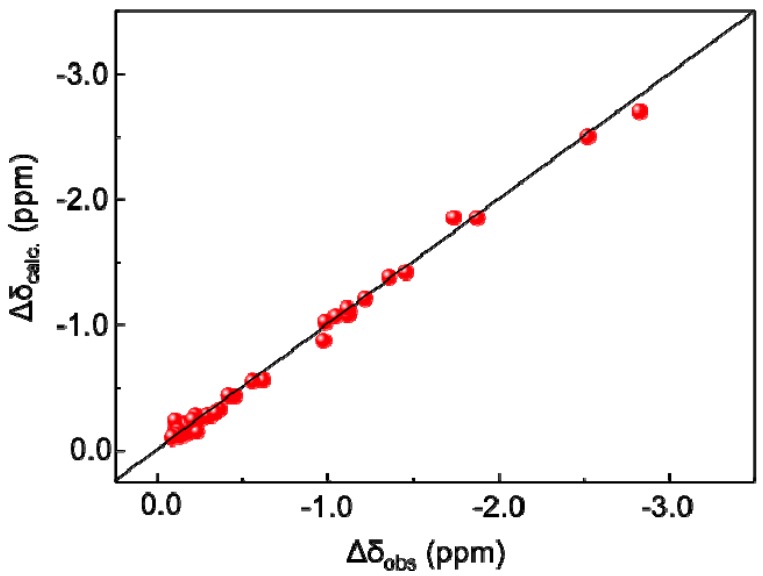
Correlations between the experimentally observed and back-calculated PCS values of the GM2 tetrasaccharide with Tm^3+^.

The PCS (∆δ) values of ^1^H and ^13^C were measured as the differences between the chemical shifts of the compound chelated to the paramagnetic ion such as Tm^3+^ and those observed with the diamagnetic ion La^3+^ in their ^1^H-^13^C HSQC spectra (Figure 2, Table 1 and Table 2). From the combined MD trajectory, 2,000 conformers of the tetrasaccharide were extracted at equal intervals to create an ensemble model, which involved transitions from a low-energy region to another in the energy landscape. According to the previously reported method [13], the PCS values of the tetrasaccharide with Tm^3+^ were back-calculated using this ensemble model. The expected PCS values were in excellent agreement with the experimental value, with a low *Q* value = 0.06 (Figure 3), which clearly validated the atomic description of this branched tetrasaccharide. *Q* = rms(∆δ_calc_ − ∆δ_obs_)/rms(∆δ_obs_). ∆δ_calc_ is given by following equation:


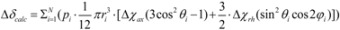


where *p_i_* is populations of each structure (set to 0.0005), *N* is number of each conformers, and (*r_i_*, *θ_i_*, *φ_i_*) defines the position vector for conformer *i* of the nucleus in polar coordinates with respect to the metal center and principal axis of Δχ tensor.

The most populated conformation of the GM2 tetrasaccharide was similar to the previously reported structure, determined by the inspection of the NOEs observed in DMSO [15] and those predicted by theoretical calculations [15,16,17,18,19]. Especially, the GalNAc–Gal moiety showed a rigid conformation with a single cluster, which is consistent with these reports. It is plausible that the bulky acetyl group of the GalNAc residue restricts the motional freedom of this glycosidic linkage. The Neu5Ac-Gal glycosidic linkage has two conformational clusters, which were similarly indicated in previous studies using Monte Carlo-based calculations [18] and an MD simulation with another force field [19].

**Table 1:** ^1^H and ^13^C chemical shifts of the GM2 tetrasaccharide tagged with La^3+^, Tm^3+^, and Tb^3+^ ions.

	La^3+^		Tm^3+^		Tb^3+^	
	Δδ^13^C/ppm	Δδ^1^H/ppm	Δδ^13^C/ppm	Δδ^1^H/ppm	Δδ^13^C/ppm	Δδ^1^H/ppm
**Glc 1**	79.95	5.132	77.12	2.620	n.d. ^(a)^	n.d.
**2**	71.44	3.547	69.57	1.811	n.d.	n.d.
**3**	75.15	3.676	73.93	2.565	n.d.	n.d.
**4**	78.06	3.652	76.94	2.670	75.97	1.683
**5**	76.63	3.652	75.17	2.295	n.d.	n.d.
**6**	59.99	3.884	58.86	2.840	58.01	2.049
**6**	59.98	3.772	58.86	2.800	58.01	1.939
**Gal 1**	102.7	4.480	102.1	3.922	101.6	3.325
**2**	70.10	3.305	69.65	2.890	69.20	2.416
**3**	74.43	4.074	74.09	3.778	73.75	3.419
**4**	77.31	4.046	77.00	3.823	76.63	3.514
**5**	74.12	3.698	73.75	3.360	73.34	2.932
**6**	60.67	3.751	60.39	3.530	60.00	3.142
**6**	60.66	3.702	60.39	3.480	60.00	2.985
**Neu5Ac 3**	36.98	2.590	36.77	2.393	36.64	2.143
**3**	36.99	1.844	36.76	1.612	36.64	1.346
**4**	68.78	3.700	68.57	3.570	68.44	3.409
**5**	51.66	3.731	51.47	3.577	51.39	3.403
**6**	73.15	3.405	72.96	3.275	72.81	3.103
**7**	68.11	3.508	67.92	3.407	67.81	3.263
**8**	72.37	3.669	72.18	3.505	72.03	3.285
**9**	62.90	3.792	62.76	3.677	62.64	3.516
**9**	62.90	3.543	62.75	3.441	62.64	3.289
**GalNAc 1**	102.8	4.680	102.6	4.480	102.3	4.227
**2**	52.40	3.845	52.17	3.641	51.97	3.324
**3**	71.38	3.594	71.20	3.470	71.02	3.278
**4**	67.88	3.844	67.71	3.747	67.54	3.564
**5**	74.82	3.637	74.64	3.524	74.45	3.315
**6**	61.23	3.693	61.10	3.609	60.93	3.405

^(a)^ Not detected due to low S/N ratio.

**Table 2:** PCS values (ppm) derived from the Tm^3+^ ion.

	Glc		Gal		Neu5Ac		GalNAc	
	Δδ^13^C	Δδ^1^H	Δδ^13^C	Δδ^1^H	Δδ^13^C	Δδ^1^H	Δδ^13^C	Δδ^1^H
**1**	−2.83	−2.51	−0.62	−0.56			−0.23	−0.20
**2**	−1.87	−1.74	−0.45	−0.41			−0.23	−0.20
**3**	−1.22	−1.11	−0.34	−0.30	−0.22	−0.23/−0.20	−0.18	−0.12
**4**	−1.12	−0.98	−0.31	−0.22	−0.22	−0.13	−0.16	−0.10
**5**	−1.46	−1.36	−0.36	−0.34	−0.20	−0.15	−0.17	−0.11
**6**	−1.12	−1.04/−0.97	−0.27	−0.22	−0.19	−0.13	−0.13	−0.08
**7**					−0.18	−0.10		
**8**					−0.19	−0.16		
**9**					−0.15	−0.11/−0.10		

### 2.2. Conformational Comparison between the GM2 and GM3 Sugar Chains

**Figure 4:**
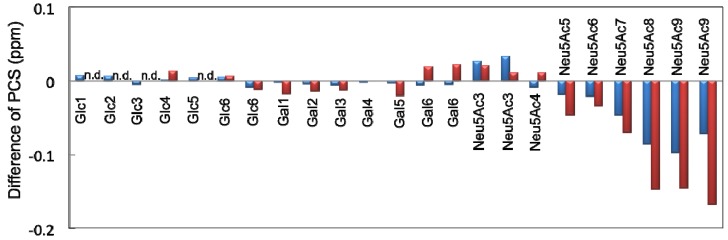
The differences of proton PCS values shown as subtraction of Δδ^1^Hof the GM2 tetrasaccharide from Δδ^1^Hof the GM3 trisaccharide with Tm^3+^ (blue) and Tb^3+^ (red). n.d., not detected due to extensive line broadening.

In a previous study, we evaluated the conformational ensemble of the GM3 trisaccharide using the same protocol [13]. To examine the impact of the additional GalNAc branch on the conformational space of the other part of the GM2 glycan, the experimental proton PCS data and the simulated glycosidic torsion angles were compared between the GM2 and GM3 sugar chains. The Neu5Ac residues exhibited significant differences in the PCS induced by Tm^3+^ or Tb^3+^; however, there were marginal differences between the Gal and Glc residues (Figure 4). These data suggest that the conformation of the sialyl linkages is different between the GM2 tetrasaccharide and the GM3 trisaccharide, whereas the conformation of the inner lactose part of the ganglioside is slightly affected by attaching the outer GalNAc residue. In agreement with the PCS data, the simulated ensemble of the Gal-Glc linkage torsion angles was very similar between the GM2 tetrasaccharide and GM3 trisaccharide (Figure 1c,e). In contrast, significant differences were observed in the simulated conformational ensemble of the Neu5Ac-Gal glycosidic linkages. In the GM3 trisaccharide, the conformations of this linkage is most populated in the cluster (Φ, ψ) = (−90° ± 11°, −57° ± 11°), while the corresponding cluster is missing in the GM2 tetrasaccharide (Figure 1b and Figure 1d). This suggests that the additional GalNAc branch restricts the conformational freedom of the Neu5Ac-Gal glycosidic linkage in the GM2 tetrasaccharide. 

**Figure 5:**
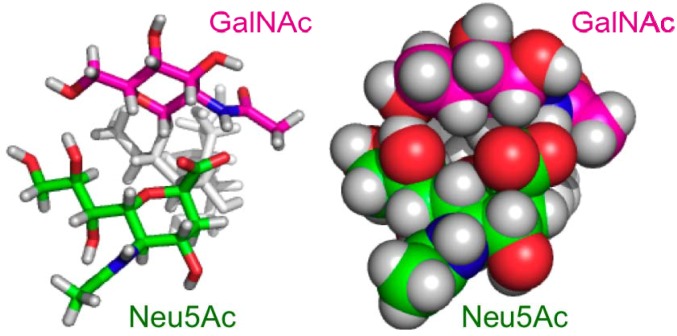
Three-dimensional structural model of one of the major conformers of the GM2 tetrasaccharide. Cylinder (**left**) and space-filling (**right**) models are shown in the same orientation.

The conformation of the Neu5Ac-Gal moiety with the torsion angles (Φ, ψ) = (−90° ± 11°, −57° ± 11°) was sterically hindered by the GalNAc residue in the branched GM2 tetrasaccharide. To avoid the steric hinderance, the torsion angles of the Neu5Ac-Gal linkage are populated in the other clusters, (Φ, ψ) = (−174° ± 11°, −32° ± 11°) and (−69° ± 10°, −6° ± 14°) in the GM2 tetrasaccharide. In the former conformational cluster, the side chain of Neu5Ac consisted of the C7, C8, and C9 groups oriented in spatial proximity to the GalNAc residue, presumably with preferable interactions between these residues, as indicated in previous reports [15,17]. For example, structural arrangements in which the hydroxyl group at C8 or C9 of the Neu5Ac residue and the hydroxyl group at C6 of the GalNAc residue are close to each other are frequently observed in the MD trajectory. In addition, atomic contacts were observed between the GalNAc amide group and the Neu5Ac carboxylate group, suggesting the transient formation of a hydrogen bond between these groups, which was observed in the DMSO solution [20] (Figure 5). Hence, the stabilization of the major conformations of the GM2 sialyl linkage can be attributed to these inter-residue interactions [21]. In the metastable conformational cluster of the GM2 sialyl linkage, a water molecule was frequently found between the Neu5Ac side chain and the GalNAc residue, maintaining the distance between the Neu5Ac carboxylate and GalNAc amide groups. The observed differences in the conformational space of the Neu5Ac-Gal linkage between the GM2 and GM3 sugar chains are qualitatively consistent with the results of the MD calculations of the sugar moieties of GM2 and GM3 employing another force field [19] and the Monte Carlo-based calculations of the GM2 headgroup and the αNeu5Ac-(2-3)-Gal disaccharide [18].

## 3. Experimental

### 3.1. General

Reagents and solvents were commercially available and used without any further purification unless otherwise noted. Column chromatography was performed using Silica Gel 60N purchased from Kanto Chemical Co., Inc., Wakosil 40C18 from Wako Pure Chemical Industries, Ltd., or Waters Sep-Pak C18. High-resolution MS measurements were performed on a JEOL JMS-777V spectrometer (Akishima, Japan). The NMR spectra were recorded on a JEOL JNM ECA-600 spectrometer equipped with a 5-mm FG/HCN probe. TMS (in CDCl_3_) served as internal standard for the ^1^H- and ^13^C-NMR measurements.

### 3.2. MD Simulations of the Sugar Moiety of GM2

All-atom MD simulations of the GM2 tetrasaccharide were employed using the Sander module of the Amber11 package [22] with the GLYCAM_06 force field. To create the topology file of the GM2 tetrasaccharide, the tLeap module of the AmberTools1.5 program was used. The initial structure was determined on the basis of preliminary MD calculations, in which the glycosidic torsion angles Φ and ψ were 39.8° and −43.6° for Gal-Glc, 42.1° and 16.9° for GalNAc–Gal, and −175.2° and −25.6° for Neu5Ac-Gal linkages. TIP3P waters were added to the solvent layer to ensure a depth of at least 8 Å from any atom and ten Na^+^ ions and nine Cl^−^ ions were added to neutralize the system. Before the MD runs were performed, the entire system energy was minimized by 500 steps of the steepest decent followed by 500 steps of the conjugate gradient. The system was heated to 300 K with a 2-fs time step in the NPT ensemble [23] at 1 atm over 50 ps using isotropic position scaling. Productive MD simulations were performed for 12 ns at 300 K with a 2-fs time step in the NPT ensemble. The initial velocities were randomized. The scaling of nonbonded 1–4 van der Waals and electrostatic interactions was not performed (*i.e.*, SCEE = SCNB = 1.0). All bonds involving hydrogen atoms were constrained using the SHAKE algorithm [24], and long-range electrostatics were treated by the particle-mesh Ewald method [25]. Snapshots were collected every 1 ps. Ten MD trajectories excluding the first 2 ns were combined into one. The analysis of the trajectories was performed using the PTRAJ module of the AmberTools1.5 program, and molecular graphics images were produced using VMD [26].

### 3.3. Preparation of the Tagged GM2 Tetrasaccharide

**Scheme 2:**
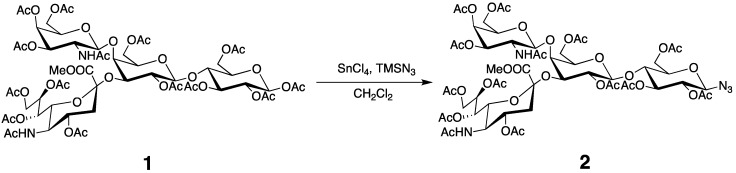
Preparation of azide **2**.

Trimethylsilyl azide (468 μL, 3.56 mmol) and SnCl_4_ (218.5 μL, 1.86 mmol) was added to a solution of compound **1** (600 mg, 0.437 mmol) in CH_2_Cl_2_ (5 mL) at 0 °C. The mixture was stirred at RT for 12 h. Then, the reaction mixture was diluted with CHCl_3_, washed with saturated aqueous NaHCO_3_, H_2_O, dried with Na_2_SO_4_, and concentrated. The residue was purified by column chromatography on silica gel (CHCl_3_/MeOH 20:1) to give **2** (528 mg, 89%) (Scheme 2). ^1^H-NMR (600 MHz, CDCl_3_, 300 K): δ = 5.98 (d, *J* = 6.90 Hz, GalNAc-NH), 5.83 (dd, *J* = 3.4, 11.0 Hz, 1H, GalNAc-H3), 5.53 (m, 1H, Neu5Ac-H8), 5.35 (m, 2H, Neu5Ac-H7, GalNAc-H4), 5.17 (t, *J* = 9.67 Hz, 1H, Glc-H3), 5.13 (d, *J* = 8.23 Hz, 1H, GalNAc-H1), 5.05 (d, *J* = 10.3Hz, 1H, Neu5Ac-NH), 4.97 (dd, *J* = 7.57, 10.3 Hz, 1H, Gal-H2), 4.86 (t, *J* = 9.26 Hz, 1H, Glc-H2), 4.81 (m, 1H, Neu5Ac-H4), 4.61 (m, 2H, Gal-1H, Glc-1H), 4.49 (m, 1H, Glc-H6a), 4.34 (dd, *J* = 2.75, 13.0 Hz, 1H, Neu5Ac-H9a), 4.22 (m, 2H, GalNAc-H6a, Gal-H3), 4.12–3.93 (m, 6H, GalNAc-H6b, Neu5Ac-H9b, Glc-H6b, Gal-H6, Neu5Ac-H5), 3.83 (m, 6H, Glc-H4, Gal-H5, Neu5Ac-H6, Neu5Ac-COOCH_3_), 3.68 (m, 1H, Glc-H5), 3.59 (t, *J* = 5.82 Hz, 1H, GalNAc-H5), 3.50 (d, *J* = 2.06 Hz, 1H, Gal-H4), 3.38 (m, 1H, GalNAc-H2), 2.81 (dd, *J *= 4.34, 13.3 Hz, 1H, Neu5Ac-H3a), 2.21–1.77 (m, 42H, OAc, NHAc ), 1.72 (t, *J* = 12.5 Hz, Neu5Ac-H3b). ^13^C-NMR (150 MHz, CDCl_3_, 300 K): δ = 171.3, 171.0, 170.3, 169.9, 168.8, 100.6, 99.25, 87.78, 75.48, 74.97, 73.54, 73.02, 72.94, 72.20, 71.92, 71.08, 70.24, 69.59, 68.62, 67.55, 66.83, 63.38, 62.27, 62.06, 61.50, 53.05, 52.93, 49.43, 37.36, 25.52, 23.20, 21.60, 20.97, 20.69. HRMS (FAB): Calcd for C_56_H_78_N_5_O_35_ [M+H^+^]: 1380.4399; Found: 1380.4484.

**Scheme 3:**
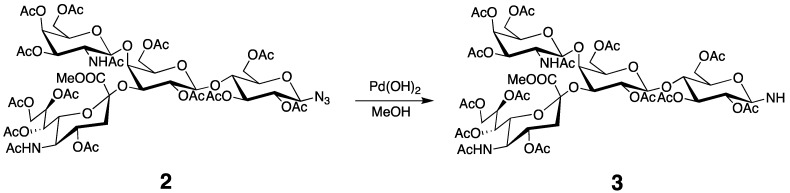
Preparation of amine **3**.

Compound **2** (528 mg, 0.383 mmol) was dissolved in MeOH (5 mL), and to this solution, Pd/C (10 mg) was added. The mixture was stirred in a hydrogen atmosphere at RT for 12 h. The reaction mixture was filtered through celite, concentrated, and then purified by column chromatography on silica gel (CHCl_3_/MeOH 20:1) to give **3** (460 mg, 78%) (Scheme 3). ^1^H-NMR (600 MHz, CDCl_3_, 300 K): δ = 6.14 (d, *J *= 6.87 Hz, 1H, GalNAc-NH), 5.88 (dd, *J* = 3.50, 11.3 Hz, 1H, GalNAc-H3), 5.51 (m, 1H, Neu5Ac-H8), 5.36 (m, 2H, Neu5Ac-H7, GalNAc-H4), 5.16 (m, 2H, Glc-H3, GalNAc-H1), 5.05 (d, *J* = 10.3 Hz, 1H, Neu5Ac-NH), 4.97 (dd, *J *= 7.72, 10.3 Hz, 1H, Gal-H2), 4.79 (m, 1H, Neu5Ac-H4), 4.73 (t, *J* = 9.30 Hz, 1H, Glc-H2), 4.57 (d, *J* = 7.96 Hz, 1H, Gal-H1), 4.44 (d, *J* = 11.4 Hz, 1H, Glc-H6a), 4.34 (dd, *J* = 2.84, 13.2 Hz, 1H, Neu5Ac-H9a), 4.21 (m, 2H, GalNAc-H6a, Gal-H3), 4.15–3.93 (m, 7H, Gal6, GalNAc-H6b, Neu5Ac-H9b, Glc-H6b, Neu5Ac-H5, Glc-H1), 3.86–3.74 (m, 6H, Neu5Ac-COOCH_3_, Gal-H5, Neu5Ac-H6, Glc-H4), 3.58 (m, 2H, GalNAc-H5, Glc-H5), 3.49 (m, 1H, Gal-H4), 3.32 (m, 1H, GalNAc-H2), 2.82 (dd, *J* = 4.57, 12.8 Hz, 1H, Neu5Ac-H3a), 2.21–1.78 (m, 42H, OAc, NHAc), 1.72 (t, *J* = 12.7 Hz, Neu5Ac-H3b). ^13^C-NMR (150 MHz, CDCl_3_, 300 K): δ = 172.1, 171.0, 170.2, 168.8, 100.5, 99.03, 84.75, 76.23, 73.84, 73.53, 72,86, 72.47, 72.23, 71.79, 70.24, 69.73, 68.70, 68.39, 67.56, 66.91, 63.39, 62.62, 62.16, 61.52, 53.23, 52.88, 49.49, 37.39, 23.57, 23.24, 21.56, 21.01, 20.72. HRMS (FAB): Calcd for C_56_H_80_N_3_O_35_ [M^+^]: 1353.4494; Found: 1354.4568.

**Scheme 4:**
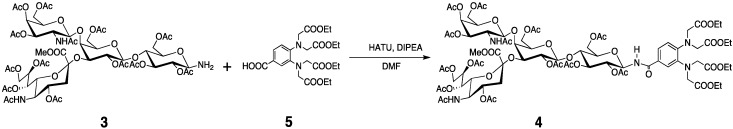
Preparation of compound **4**.

Compound **5** (250.8 mg, 0.5 mmol) [13], DIPEA (1 mL, 5.7 mmol), and HATU (240 mg, 0.62 mmol) were dissolved in DMF (5 mL), and the mixture was stirred at RT for 10 min. Subsequently, the mixture was transferred to a solution of compound **3** (400 mg, 0.30 mmol) and DIPEA (100 μL, 0.57 mmol) in DMF (1 mL), and this solution was stirred at RT for 12 h. The reaction mixture extracted with EtOAc was washed with H_2_O, dried with Na_2_SO_4_, and concentrated. The residue was purified on a silica gel column with CHCl_3_/MeOH (25:1) to give **4** (232 mg, 43%) (Scheme 4). ^1^H-NMR (600 MHz, CDCl_3_, 300 K): δ = 7.51 (d, J = 2.08 Hz, 1H, Ar-H), 7.26 (1H, Ar-H), 6.99 (d, *J* = 8.24 Hz, Ar-H), 6.83 (d, *J* = 9.02 Hz, 1H, CONH), 6.21 (d, *J *= 6.88 Hz, 1H, GalNAc-NH), 5.9 (dd, *J* = 3.43, 11.0 Hz, 1H, GalNAc-H3), 5.49 (m, 1H, Neu5Ac-H8), 5.39 (dd, *J* = 2.76, 9.62 Hz, 1H, Neu5Ac-H7), 5.34 (d, *J* = 3.43 Hz, 1H, GalNAc-H4), 5.31 (m, 2H, Glc-H3, Glc-H1), 5.16 (d, *J *= 8.13 Hz, 1H, GalNac-H1), 5.05 (d, *J* = 10.3 Hz, 1H Neu5Ac-NH), 4.96 (m, 2H, Gal-H2, Glc-H2), 4.82 (m, 1H, Neu5Ac-H4), 4.56 (d, *J* = 8.23, 1H, Gal-H1), 4.44–4.19 (m, 12H, Glc-H6a, Neu5Ac-H9a, GalNAc-H6a, Gal-H4, tag-NCH_2_), 4.1 (m, 11H, Glc-H6b, GalNAc-H6b, Gal-H6a, COOCH_2_), 4.04–3.93 (m, 3H, Neu5Ac-H5, Neu5Ac-H9b, Gal-H6b), 3.86–3.74 (m, 7H, Glc-H4, Glc-H5, Gal-H5, Neu5Ac-H6, Neu5Ac-COOCH_3_), 3.60 (t, *J* = 6.00 Hz, 1H, GalNAc-H5), 3.50 (d, *J* = 2.04 Hz, 1H, Gal-H4), 3.33 (m, 1H, GalNAc-H2), 2.83 (dd, *J* = 4.15, 13.1 Hz, 1H, Neu5Ac-H3a), 2.21–1.83 (m, 42H, OAc, NHAc), 1.74 (m, 1H, Neu5Ac-H3b), 1.18 (m, 12H, tag-CH_3_). ^13^C-NMR (150 MHz, CDCl_3_, 300 K): δ = 173.4, 172.1, 170.9, 170.0, 168.5, 167.1, 163.1, 145.9, 141.6, 127.7, 122.1, 121.7, 120.8, 100.4, 99.05, 79.00, 75.72, 74.56, 73.56, 72.83, 72.70, 72.20, 71.77, 71.08, 70.25, 69.74, 68.71, 68.35, 67.59, 66.91, 63.39, 62.07, 61.54, 60.72, 53.30, 52.92, 52.22, 49.50, 37.32, 23.50, 23.24, 21.59, 20.98, 20.78, 14.19. HRMS (FAB): Calcd for C_79_H_110_N_5_O_44 _[M+H^+^]: 1832.6445; Found: 1832.6515.

**Scheme 5:**
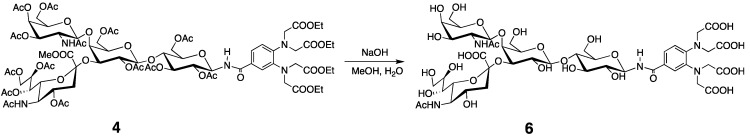
Preparation of the GM2 tetrasaccharide with the tag.

Compound **4** (100 mg) was dissolved in MeOH and small aliquots of a 1 M NaOH aqueous solution were added until the reaction was complete (checked by TLC). The reaction mixture was purified on an ODS column to give **6** (52 mg, 79%) (Scheme 5). ^1^H-NMR (600 MHz, D_2_O, 300 K): δ = 7.29 (m, 2H, Ar-H), 6.79 (d, *J *= 8.77 Hz, 1H, Ar-H), 5.08 (d, *J* = 9.36 Hz, 1H, Glc-H1), 4.66 (1H, GalNAc-H1), 4.47 (d, *J* = 7.68 Hz, Gal-H1), 4.02–4.12 (m, 6H, NHCH_2_, Gal-H3, Gal-H4), 3.91 (s, 4H, NHCH_2_), 3.86–3.82 (m, 3H, GalNAc-H4, Glc-H6a, GalNAc-H2), 3.78 (m, 1H, Neu5Ac-H9a), 3.74 (m, 2H, Glc-H6b, Gal-H6a), 3.71–3.66 (m, 7H, Gal-H6b, GalNAc-H6, Neu5Ac-H5, Neu5Ac-H4, Neu5Ac-H8, Gal-H5), 3.62 (m, 4H, GalNAc-H5, Glc-H3, Glc-H5, Glc-H4), 3.58 (dd, *J* = 3.40 Hz, 10.7, 1H, GalNAc-H3), 3.55–3.48 (m, 3H, Neu5Ac-H9b, Neu5Ac-H7, Glc-H2), 3.38 (d, *J* = 10.6 Hz, Neu5Ac-H6), 3.29 (t, *J* = 8.86 Hz, Gal-H2), 2.57 (dd, *J* = 4.02, 12.7 Hz, Neu5Ac-H3a), 1.93 (6H, NHAc), 1.83 (t, *J *= 11.7 Hz, 1H, Neu5Ac-H3b). ^13^C-NMR (150 MHz, D_2_O, 300 K): 182.2, 179.8, 175.6, 172.3, 146.1, 140.3, 124.4, 121.9, 119.6, 118.4, 102.8, 80.07, 78.20, 77.06, 76.51, 74.93, 74.38, 73.30, 72.15, 71.55, 69.97, 68.89, 68.29, 67.85, 62.89, 61.09, 60.06, 54.61, 52.49, 51.89, 37.20, 22.43.

### 3.3. PCS Observation and Analyses of the GM2 Tetrasaccharide

Compound **6** (2.4 mg) was dissolved in D_2_O (0.6 mL) and the pH was increased to 8.0 by adding a solution of NaOD. This solution was titrated with a D_2_O solution of MCl_3_ (250 mM; M = La^3+^, Tm^3+^, or Tb^3+^) for the NMR measurements. For the PCS observations, ^1^H-^13^C HSQC spectra were recorded at 300 K with 512 (*t*_1_) and 1024 (*t*_2_) complex points. The NMR spectra were processed and analyzed with the NMRPipe [27] and Sparky [28] programs.

Two thousand conformers were extracted from the combined trajectory of the GM2 tetrasaccharide every 50 ps, and the averaged paramagnetic center defined from additional MD calculations of the tag moiety [13] was added by aligning each glucose ring. A single Δχ tensor was determined for the conformational ensemble by inspection of the experimentally obtained PCSs with the assumption that every conformer contributes equally to the PCSs by a modified version of MSpin [29].

## 4. Conclusions

In summary, the lanthanide-assisted NMR approach was successfully applied to the characterization of the conformational dynamics of the branched sugar moiety of GM2. The conformational space of this tetrasaccharide predicted from MD calculations was successfully validated on the basis of the PCS data of the ensemble model. In addition, the interbranch interactions that are responsible for the conformational differences between the GM2 tetrasaccahride and the GM3 trisaccharide were identified by the paramagnetic NMR method in conjunction with MD simulations. The success of our systematic approach opens new prospects for the conformational analysis of dynamic structures of more complex, high-antennary oligosaccharides toward decoding glycocodes from 3D structural aspects.
